# Improving Sierra Leone’s skilled health-worker-to-population ratio: how unsalaried and auxiliary health workers are barriers in its path to universal health coverage

**DOI:** 10.1136/bmjgh-2025-021043

**Published:** 2025-11-17

**Authors:** Pieternella Pieterse, Federico Saracini

**Affiliations:** 1 Institute of Global Surgery, Royal College of Surgeons in Ireland, Dublin, Ireland

**Keywords:** Health systems, Global Health, Health Personnel, Health education and promotion, Health Services Accessibility

## Abstract

**Background:**

Achieving Universal Health Coverage (UHC) is one of Sierra Leone’s main health policy goals. To achieve UHC, a country needs a skilled-health-worker-to-population ratio of 44.5 doctors, midwives and nurses per 10 000. In Sierra Leone, this ratio is 6.4 per 10 000. There is limited government funding to expand the health worker payroll, and the majority of healthcare providers on the payroll are auxiliary cadres, who fall below WHO’s definition of ‘skilled’ health workers.

Since 2010, approximately 10 000 auxiliary nurses have been engaged in the public health system on an unsalaried ‘volunteer’ basis. They compete for paid employment with graduates who meet skilled health worker criteria. This study examines barriers and enablers to Sierra Leone’s expansion of its skilled health workforce.

**Methods:**

Mixed methods: trainee health worker (projected) enrolment data were collected for 2019–2027. Primary care facility staffing data at district level was collected in 2023–2024, salaried and unsalaried. Semistructured interviews were conducted with students, faculty (n=20), health workers (n=110), salaried and unsalaried staff and key informants. The health labour market framework for UHC was used to analyse the results.

**Results:**

Since 2019, Sierra Leone’s emphasis has shifted to training nurses who meet WHO standards. This has led to a significant increase in public and private institutions offering health worker training courses. In 4 years, enrolment in nursing training facilities has quadrupled. District level data show that, at primary care level, over 50% of public health workers are in unsalaried positions, waiting for paid public employment.

**Conclusion:**

While the production of additional health workers can be a potential enabler to a health worker density improvement, a lack of government funding to absorb both new graduates and all trained volunteer health workers who have been waiting for job opportunities means that barriers to a health workforce expansion outweigh the enablers.

**WHAT IS ALREADY KNOWN ON THIS TOPIC**

- Many African countries face paradoxical health worker surpluses. Limited public budget to hire healthcare staff, despite significant needs-based shortages, leaves many health workers unemployed, especially when health worker training capacity expands beyond absorption capacity.

**WHAT THIS STUDY ADDS**

- Sierra Leone has been informally recruiting ‘surplus’ health workers as unsalaried volunteers since 2010. This has resulted in over half of its primary healthcare staff, predominantly auxiliary nurses, working without pay, waiting for salaried employment. This backlog slows the absorption of recently graduated skilled health workers, delaying UHC achievement and creating barriers to access to care.

**HOW THIS STUDY MIGHT AFFECT RESEARCH, PRACTICE OR POLICY**

- Many other low-income countries with high health worker unemployment may also employ informal arrangements to retain their ‘surplus’ health workforce. More research is needed into whether, and where this practice is common, and what its effects are.

## Background

### Universal health coverage in low-income countries

 Health for All is the third of the Sustainable Development Goals (SDGs), which aims to ‘ensure healthy lives and promote well-being for all, at all ages’.^1^ The inclusion of Universal Health Coverage (UHC) as SDG subtarget 3.8 has been particularly relevant to low-income countries (LICs) as its objective is to ensure that all people can access the health services they need, without facing financial hardship.^1^ UHC achievement is verified by a country’s ‘Essential Health Services Coverage Index’ score, including service capacity, health workforce and access to care.^2^ Making progress towards UHC is not only beneficial to the health outcomes of a country’s population, it also determines a country’s inclusion on the WHO health workforce support and safeguard list, as it uses UHC scores as one of its inclusion criteria.^3^

Countries have their own roadmaps for achieving UHC. Several studies have assessed which interventions can most improve UHC scores.^4^ A study by Reid *et al* demonstrated that, based on a dominance analysis assessing 183 countries, increasing the density of the health workforce created the largest UHC impact.^5^ However, achieving greater health workforce density can be challenging for LICs with limited health budgets.

### Sierra Leone case study

Sierra Leone, a small West African country with a population of over 8 million, aims to achieve UHC by 2030, according to its National Health and Sanitation Policy 2021.^6^ In 2021, its UHC progress was scored at 41 out of 100.^7^ The country’s health system was badly affected by the 1991–2002 civil war,^8^ by the Ebola epidemic, 2014–2016, and, to a lesser extent, the COVID-19 pandemic.^9 10^ The country’s health outcomes remain poor. Maternal mortality rate has improved significantly in the past decade, but remains ranked among the worst worldwide, along with Sierra Leone’s under-five, infant and neonatal mortality.^11^ As the country’s skilled-health-worker-to-population ratio stands at 6.4 per 10 000 population, achieving the UHC target of 44.5 per 10 000 is still some way off.^12^

Sierra Leone’s health sector depends heavily on out-of-pocket spending, which finances an estimated 55% of total cost.^13^ Until the 2025 changes in the aid financing landscape, donor spending accounted for about 26% of health expenditure.^14^ The country’s health financing trends have been stagnant. In 2024, the Government of Sierra Leone (GoSL) had planned to spend 7% of its budget on health, with 67% of that earmarked for salaries within the health sector.^15^ Without greater financial prioritisation of the health sector, there is limited scope to allocate additional funding towards achieving UHC.^14^ Sierra Leone’s health policy and strategy documents highlight the need for ‘skilled health workers’, as defined by WHO, to meet UHC target.^16 17^ However, this is a tall order: the current payroll provides salaries for approximately 18 000–20 000 staff and only around 5500 staff are skilled health workers.^18^

Sierra Leone’s 2025 health workforce continues to be shaped by its postwar health worker shortage. From 2002 onwards, large numbers of Maternal and Child Health Aides (MCHA) were trained at free training facilities in every district, funded by UNICEF. State-Enrolled Community Health Nurse (SECHN) training became the most popular ‘nursing’ course at that time. Neither SECHNs nor MCHAs meet WHO Skilled Health Worker minimum standards.^19^ In 2019, GoSL instructed training facilities to pause MCHA and SECHN training programmes, due to overproduction^20^; at that stage, an estimated 43% of Sierra Leone’s healthcare providers were SECHNs, and 25% were MCHAs.^21^ While emphasis quickly shifted towards training ‘skilled’ health workers in 2019, there appears to have been little discussion, even less policy engagement, regarding ‘upskilling’ the 12 000+ predominantly SECHN and MCHA cadre health workers on the payroll, who do not meet the global minimum standard.

### So-called volunteer health workers

The 2016 Sierra Leone human resources for health (HRH) audit report was the first to confirm that almost 10 000 ‘volunteer’ workers were engaged within the public health sector.^22^ While some volunteers were cleaners and porters, 36% of the total clinical staff deployed in Sierra Leone’s public sector were unsalaried volunteer staff, mostly MCHAs and SECHNs.^20^ Its origins go back to 2010, when Sierra Leone reformed its primary healthcare system and introduced free healthcare services for pregnant and lactating mothers and children under five years.^23^ The health policy overhaul led to the recruitment of 1000+ additional health workers. Many had allegedly been working in public facilities as self-charging ‘volunteers’, which had been common practice during the civil war.^24^ After 2010, district officials started to routinely deploy MCHA and SECHN graduates, with a promise that there would soon be a chance for them to be added to the centralised payroll.^25^ This system appears to have proceeded unchecked ever since, creating an increasingly large cohort of unsalaried health workers who are waiting to apply for pay-rolled jobs.^16^ Since the 2016 HRH audit revealed the extent of the volunteering, both Sierra Leone’s MoH and the WHO have suggested pathways to eliminate the presence of trained health workers without a contract or salary from public health facilities, but the presence of volunteer clinical staff continues to be a prominent feature of the country’s health system.

This paper examines the barriers and enablers that Sierra Leone faces to increase its health workforce density to achieve SDG goal 3.8. While this is largely a health-financing related issue, this study examines whether the country’s large share of unsalaried volunteer health workers creates an unusual blockage that further delays the absorption of much needed skilled health worker graduates into the public sector workforce.

## Materials and methods

This is a mixed methods study, using quantitative primary data collected from accredited health worker training facilities in Sierra Leone, and staffing data collected from District Health Management Teams (DHMTs) in 2024. All training facilities and DHMTs were contacted by phone and email with the request to fill in the data collection sheet (onlinesupplemental files 1  2). A cover note explaining the research objectives and copies of the research ethics approval documents were enclosed. Four follow-up calls and texts were sent between November 2023 and May 2024.

Qualitative data included semistructured interviews conducted with health worker trainees, students and faculty members (n=20), with key informants (n=15) and health workers (n=110), both salaried and unsalaried staff. Data were collected in Port Loko and Bo District and Freetown in 2023 and 2024, and research ethics approval was obtained by the researchers’ own university and from the Sierra Leone Ministry of Health and Sanitation (number 031/03/2023, obtained in March 2023 and extended in March 2024 for 1 year). Table 1 outlines the sampling considerations.

**Table 1:** Sampling considerations for semistructured and key informant interviews

Sampling for qualitative research, semi-structured interviews			
Location	Diversity considerations	Sublocation/consideration	Total interviews
Port Loko district	Rural Northern district Ethnic diversity (mainly Temne) Mainly opposition affiliated	Clusters of health facilities/communities in northern, eastern, southern and western direction from district capital Mix of ‘along tarmac road’ and ‘off road’ Mix of small, medium, large clinics Include district hospitals Include private clinics if possible Interview community members in clinic catchment areas	Health workers, all cadres, salaried and unsalaried: n=110 Community members, entitled to Free Healthcare for themselves or on behalf of their child under five years: n=66
Bo district	Rural/semi-rural Southern District Ethnic diversity (mainly Mende) Mainly ruling party affiliated		
Western area urban/freetown (capital)	Urban/slum Western/capital city Mixed ethnicities Mixed party affiliations	Better off/ urban slum areas Mix of primary care public health, public hospital, private clinic/hospital+nearby communities Interview community members in catchment areas	
**Key informant interviewees**			
Health worker training institutions in all three districts: Proprietors/managers/lecturers Students			n=20
Topic experts and stakeholders Local NGO staff in all three districts International NGO working in the health sector Aid donor representatives working in health sector			n=24

NGO, non-government organisation.

All qualitative data were audio-recorded and analysed using Lumivero Nvivo V.15 software. A thematic analysis based on Braun and Clarke^26 27^ was performed to assess what patterns and key themes could be detected within the interview data.

The study’s qualitative data were collected before the quantitative data, as it was initially assumed that the quantitative data would have been available from MoH. As these data were deemed crucial for putting the qualitative findings into context, the study design was broadened to a mixed method study after the start of the initial data collection—the research ethics approval and research budget had been designed to allow for this. Integration of both methods occurred during the analysis of the findings. Relevant quantitative and qualitative data have been presented side by side in this paper’s results and analysis sections, where data from both methods are brought together under each heading. A completed checklist to demonstrate ‘Good Reporting of Mixed Methods Studies’ and the authors’ reflexivity statement can be found as onlinesupplemental files 3  4.

**Figure 1:**
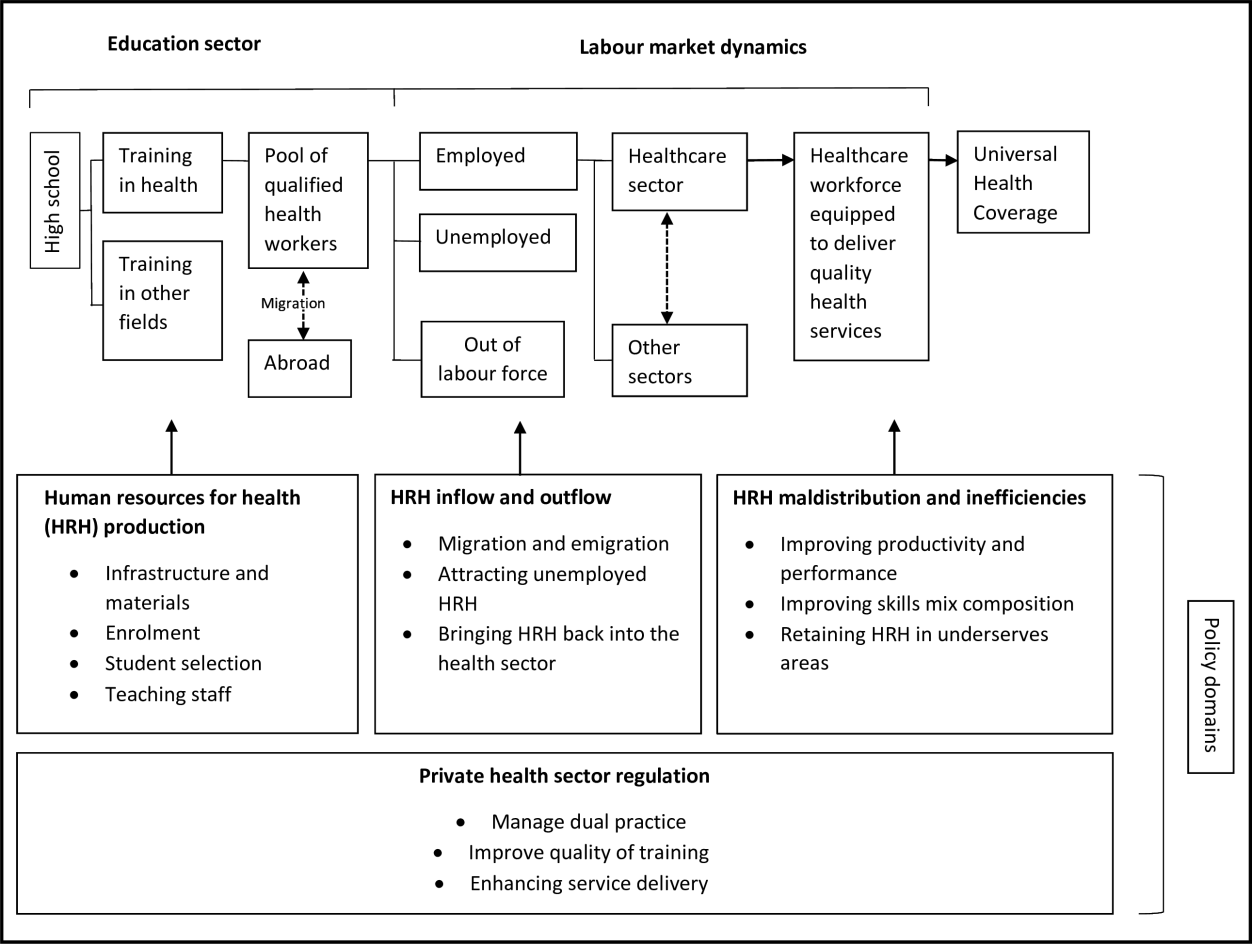
Health labour market framework to universal health coverage.^28^

A wide range of secondary quantitative and qualitative data on health policies, healthcare expenditure, health workforce data, population and the latest health labour market assessment were also consulted for this study. The health labour market framework for UHC^28^ was used to analyse the data (see figure 1), to examine how the barriers and enablers identified by our study, may impact Sierra Leone’s opportunities to address its skilled-health-worker-to-population ratio.

## Results

### State registered nursing course enrolment

A total of 10 out of 12 of Sierra Leone’s accredited health worker training facilities responded to the request for enrolment data, see table 2.

**Table 2:** Sierra Leone’s accredited providers of health worker training (excluding midwifery-only colleges), 2023–2024

#	Nursing/midwifery training facility	Included/excluded in study
1	ATMA (Kissi Brook), Freetown	Excluded (non-responsive)
2	Bu Ali Sina, Freetown	Included
3	COMAHS, University of Sierra Leone, Freetown	Included
4	Ernest Bai Koroma, Tonkolili	Included
5	Kenema town, Kenema	Excluded (non-responsive)
6	Masanga, Tonkolili	Included
7	Matru, Bonthe	Included
8	Njala University, Bo	Included
9	St John of God’s, Port Loko (affiliated to UNIMAK)	Included
10	Port Loko Nursing School, Port Loko	Included
11	UNIMAK (Makeni location only)	Included, offers no SRN training
12	University of Lunsar, Port Loko	Included

COMAHS, College of Medicine and Allied Health Sciences; SRN, state registered nursing; UNIMAK, University of Makeni.

**Figure 2:**
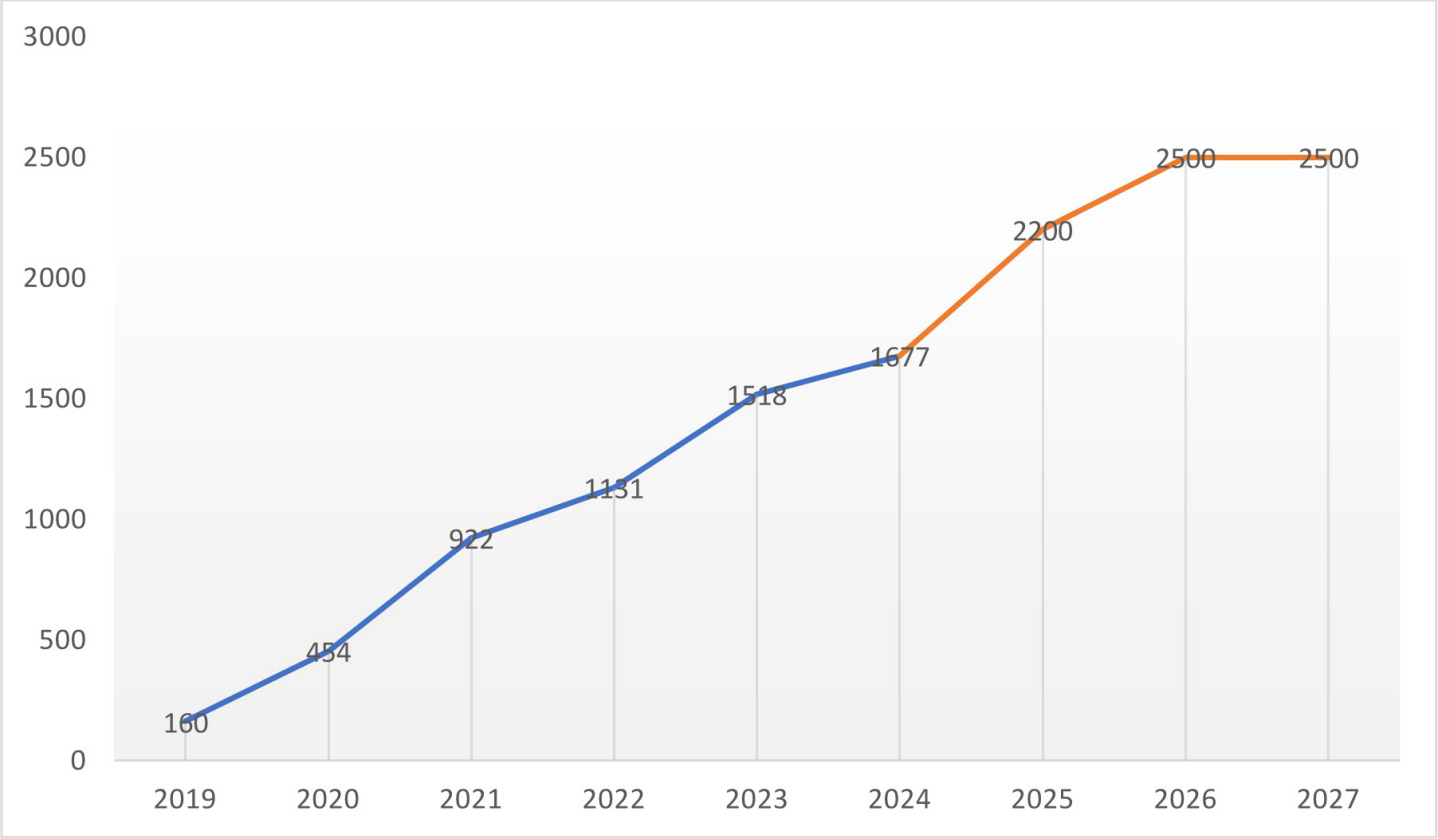
Total number of students admitted to an SRN course in Sierra Leone (from 2025 onward=projected admissions). Excludes admission figures from 2 out of 12 SRN training facilities, as requests for data went unanswered. All data were collected by, or on behalf of, the authors between October 2023 and May 2024.

From 2019 onwards, when the pause in training MCHA and SECHN cadres was introduced, courses for higher-cadre nursing and midwifery became popular. In 2023–2024, Sierra Leone had twelve accredited training facilities that provided health worker training and out of those, 11 offered state registered nursing (SRN) diploma courses, according to interviews with a MoH representative (KII006). While some training institutions offered degree courses, the rapid increase in courses for skilled health worker cadres is best illustrated by the increase in SRN diploma courses (figure 2). Only two training institutions provided SRN training prior to 2019, seven started admitting small numbers of SRN trainees in 2020 or later, and no data were available for the unresponsive two training facilities. Data shared about their intended intake of students between 2025–2027 demonstrate that all health worker training facilities were planning to increase their annual SRN student cohorts further. The two non-responsive institutions are one large and one small training facility, similar to the 10 responsive institutions, of which half were small and the other half large facilities (training approximately 100 students and 200–300 students per annum as SRNs, respectively). The combined number of additional SRN students enrolled in these two study locations is likely to have been approximately 400 per year between 2022–2024. It is therefore likely that the total number of new SRN diploma students may have already risen above 2000 per annum by 2024.

The figure 2 graph is an indication of the current trend regarding health worker ‘production’ in Sierra Leone, which is growing rapidly and appears disconnected from the MoH’s capacity to absorb graduating healthcare staff. This trend was further confirmed during an interview with the new director of HRH at the MoH, who shared in January 2025 that six additional health worker training facilities were accredited by the relevant authorities towards the end of 2024.

Interviews conducted in 2023–2024 with the proprietors or directors of the health worker training facilities revealed a mix of responses regarding their students’ paid employment opportunities on graduation. While some claimed that past pupils had quickly found themselves paid public employment, others admitted that graduates worked without salaries for up to 4–5 years (eg, TC007). Some proprietors mentioned that exploring opportunities in the private sector was becoming more attractive, even in rural Sierra Leone (TC004), or that past graduates had gone to work in the USA or UK (KII010). One senior nursing school manager was quick to quote the UHC target of 44.5 skilled health workers per 10 000, to justify the significant expansion of his institution’s student intake. He suggested that Sierra Leone needed to train over 35 000 additional skilled health workers to achieve UHC. To the question whether these trained health workers would be counted by the WHO if they were not in paid employment, he shrugged his shoulders and responded that this was not up to him to decide (TC008).

### Unsalaried health workers in primary healthcare

Senior HRH staff within the MoH, interviewed in 2024 and 2025, were unable to share data regarding how many clinically trained health workers were engaged on a voluntary basis to work in peripheral health units (PHUs, public primary healthcare facilities) and hospitals (KII006 and KII011). To verify whether the proportion of unsalaried health workers had increased, decreased or remained the same since the last study carried out in 2016,^22^ all 16 DHMTs were contacted with a request to share the clinical staffing data for all of the PHUs within their district, based on cadre and whether staff were on the payroll or not. In total, 10 out of 16 DHMTs responded to our request for data. These 10 districts were home to 55% of Sierra Leone’s total population, according to the 2021 census data,^29^ and include a mix of districts with larger urban centres, such as Western Area Urban and Bo District, and smaller, rural districts like Pujehun, which, together, are a representative sample of the entire 16 districts that make up Sierra Leone. On this basis, we suggest that it is likely that the percentage of the total number of salaried and unsalaried PHU-based health workers throughout Sierra Leone is similar to that of the 10 that shared their staffing data.

**Figure 3:**
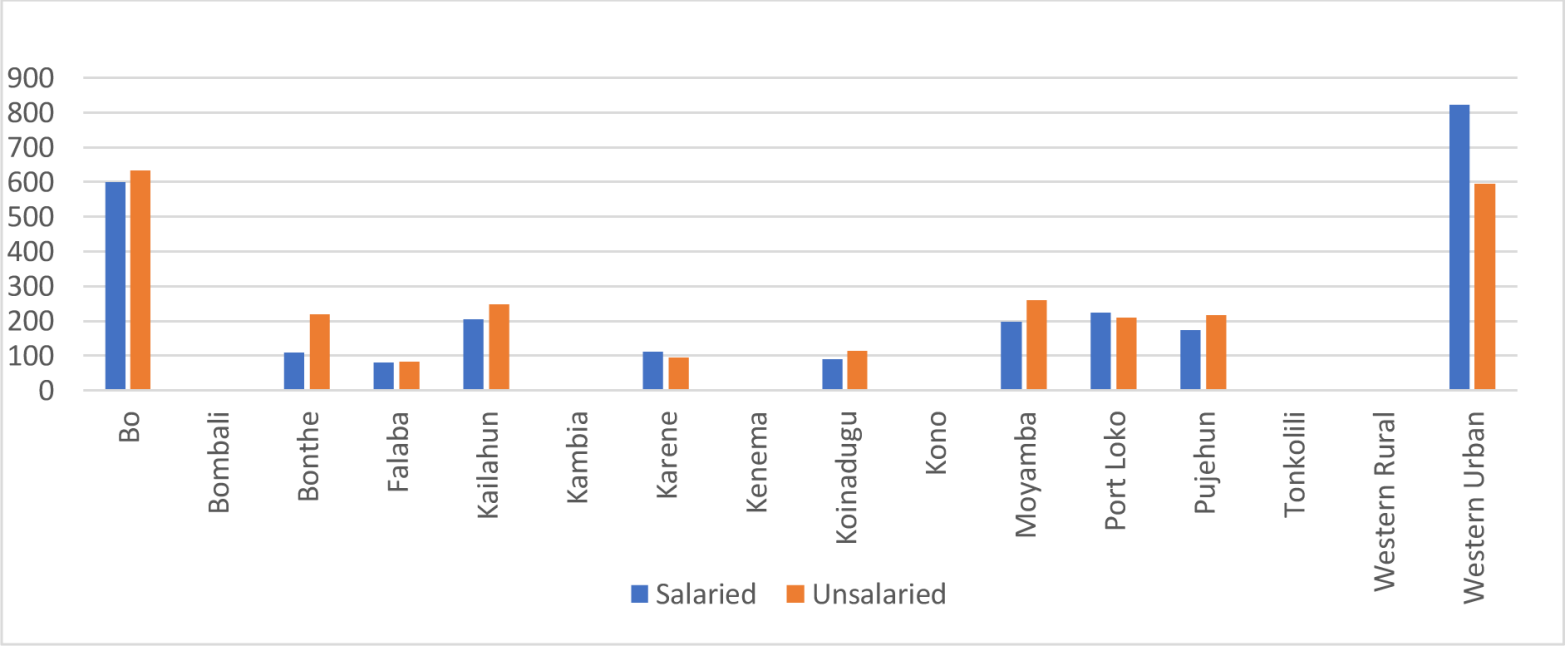
Primary health facility clinical staff per district, salaried/unsalaried, 2023–2024.

Figure 3 is a graphic depiction of the staff per district. The total number of staff on the payroll in the 10 districts was 2613, and the total number of staff who were not on the payroll (and thus unsalaried) was 2672, which means just over half (50.6%) of the total number of clinical staff at primary care facilities in these 10 districts were unsalaried.

### Qualitative data

The qualitative data collection provided the following key insights. Several key informants confirmed that at district level, the DHMT routinely offered all MCHA graduates and often also all graduate SECHN placements within PHUs and the district hospital(s) on graduation, after they had completed their 1-year ‘postbasic’ mandatory internship (DHMT003). Another DHMT key informant confirmed that recent SRN graduates were posted as volunteers to the public hospitals in the district, as this cadre is generally considered to be best suited to hospital deployment only (DHMT010). These placements, both interviewees confirmed, were accepted based on the understanding that volunteers could apply for salaried posts after completing a certain period of time volunteering. One senior MoH interviewee (KII011) was able to confirm that the Public Service Commission, which processes health worker employment applications, only accepts applications from volunteers who have completed at least 2 years of unsalaried work at a public health facility.

## Discussion

The Health Labour Market Framework, created by Sousa *et al*,^28^ has been used to analyse the barriers and enablers that influence Sierra Leone’s drive to achieve greater health workforce density and, with it, improve its UHC score. The framework (figure 1) provides a ‘comprehensive picture of health labour market dynamics and of the contributions of four groups of health workforce policies to the attainment of equitable access to quality health services and UHC’.^28^ It focuses on the following policy domains: (1) HRH production; (2) HRH inflows and outflows; (3) HRH maldistribution and inefficiencies and (4) regulation of the private health sector. While recognising that policies alone do not always address the problems they have been created to target, due to the wide policy-implementation gap^30^ that is common in many LIC contexts, the policy domains are relevant and have been used to analyse the quantitative and qualitative study data, to establish in which of these domains the findings point to barriers or enablers regarding UHC attainment.

### The production of health workers

The training enrolment survey shows a significant increase in enrolment of SRN students year-on-year, which demonstrates that, from a human resources production perspective, Sierra Leone has the potential to improve its health worker density, which is a clear enabler. A recent study focusing on 47 countries in the African Region demonstrated that significant HRH shortages exist across the continent; only four countries had densities of physicians, midwives and nurses of 44.5 per 10 000 population or above.^31^ However, as Sousa *et al*^28^ stress ‘[HRH production policies]…will succeed in producing enough health workers to fulfil the needs of the population *only if* they are designed in parallel with policies to ensure the *absorption* of new graduates into the labour market and to correct workforce maldistribution and inefficiencies’ (2014, p 893, emphasis added).

Presently, a clear barrier to the success of its HRH production is Sierra Leone’s limited healthcare financing, which means that the absorption of new graduates into the labour market is severely delayed.

### Health worker inflows and outflows

The rate at which Sierra Leone can achieve an improvement in its skilled-health-worker-per-population ratio is primarily determined by the ability to recruit additional skilled health workers (nurses with diplomas (SRNs) or degrees, midwives or doctors. This, in turn, is based on the number of additional jobs that the MoH is able to create and fund (with sanction from the Ministry of Finance) to gradually expand the health workforce.^17^ The pace at which new, skilled healthcare staff can be hired is slowed down by the fact that as many as 10 000 unsalaried volunteers, predominantly MCHAs and SECHNs auxiliary cadres, are vying for the same job opportunities. For each newly recruited health worker, MoH is in effect making a choice between hiring a skilled health worker no more than 2 years or 3 years qualified, who may not have volunteered for long, if at all; and clearing a backlog of promises to the volunteer auxiliary health workers, who may have worked for 4 years or more without a salary.

This is a significant and unusual barrier. Volunteers accepted their unsalaried assignments based on the assumption that they would get an opportunity to apply for a public sector post and get a payrolled position. Volunteer MCHAs or SECHNs did not anticipate having to compete for jobs with recent graduates who have better qualifications that count towards the country’s UHC targets. Sierra Leone would benefit from a government (or donor-funded) initiative to upskill all already payrolled SECHN and MCHAs. This would improve the quality of healthcare delivery and create a large number of skilled health workers without having to recruit additional staff. However, upskilling as many as 8000–10 000 auxiliary staff would be costly and would take a significant share of payrolled staff out of frontline service for several years during study. The fact that thousands of already skilled, self-funded SRNs (and health workers of other skilled cadres) are available to recruit without the government having to pay for their training, makes this a challenging policy proposal. Similarly, the recruitment of MCHAs or SECHNs volunteers who still require upskilling is an equally illogical step for MoH to take, if it were not for the fact that a tenuous social contract exists between the MoH and these health workers, who have provided critical frontline health services without being remunerated for years.

As far as HRH outflow is concerned, the number of Sierra Leone health workers who migrate abroad for work is thought to be limited, but no data exist. Medical doctors are more likely to be able to take up jobs in middle- or high-income countries. For nurses, midwives or auxiliary staff, the country’s inclusion on the WHO safeguard list is thought to make it difficult for individuals to secure jobs abroad, as high-income countries are discouraged from recruiting healthcare staff from countries included in the list.^3^

### Addressing HRH maldistribution and inefficiencies

The complete absence of data at MoH level regarding the number, cadre and place of deployment of the approximately 10 000 unsalaried health workers engaged across the public health system is a significant barrier to its ability to address maldistribution and HRH inefficiencies within the health system. Creating a holistic understanding of all HRH data, by adding volunteer cadres to Sierra Leone’s digital HRH information system, could be a first step to acknowledging and integrating this cohort.

The fact that a great number of health workers have been willing to carry out their profession in remote and rural locations without a salary is a potential enabler. With additional training to bring all health workers up to skilled health worker level and the implementation of the policy proposals to absorb volunteers onto the payroll, the currently unsalaried cohort could enable a much-needed improvement of Sierra Leone’s rural health services,^12 32^ if it were not for funding constraints.

The high number of students engaged in SRN courses is something that urgently needs to be examined in greater detail. This issue is a significant challenge; not only in a Sierra Leone context, but Africa-wide. It points to inefficient use of personal and/or government resources invested in training, if there are no employment prospects.^31^ Sierra Leone’s MoH has repeatedly committed to introducing workforce planning in policy papers since 2017, to ensure that education opportunities are created for approximately the right number and type of graduates that are required and funded.^6 16 33^ From a health systems and a UHC perspective, this measure cannot be introduced soon enough.

### Regulation of the private sector

This policy domain is underdeveloped in Sierra Leone and its regulatory absence can only be seen as a barrier to achieving UHC. The potential for a positive contribution of the private sector in healthcare provision, if well-regulated and enforced, has been demonstrated in a number of studies.^34 35^ However, these studies also point to the risk of a lack of regulation, which is widespread in the region. Sierra Leone’s health policies do not elaborate on private health sector regulation; they merely state that efforts should be made to engage with the private sector and establish public-private partnerships.^16^

## Study limitations

This study focused on the impact of the increasing health worker training opportunities and the number of unsalaried health workers in primary care in Sierra Leone. Regarding the training opportunities: Two out of 12 teaching institutions did not respond to repeated requests for information about their student enrolment. The data collection did not cover course completion rates; therefore, the data on the approximate number of graduates per annum remain an estimate, sufficient only to understand the approximate magnitude of the student cohort and current trajectory.

The data collection sheets on salaried and unsalaried health workers at district level were completed by 10 out of 16 DHMTs. This meant that it was impossible to calculate the exact number and cadre of all clinical health workers awaiting payroll inclusion at district level. Having data on 10 districts, where a total of 55% of the population resides, provided sufficient data to be illustrative for the purpose of our study. Due to time and financial constraints, the authors were unable to gather workforce data from all hospitals in the country.

## Conclusions

Sierra Leone’s policy aim to achieve UHC by 2030 is constrained by many factors, most notably a lack of sufficient healthcare finance to expand the health workforce. The tension that arises from having a significant pool of trained and educated youth seeking employment and the overall lack of job opportunities is a continent-wide phenomenon.^36^ Using payroll deferrals as a temporary measure to compensate for domestic health worker shortages and retain trained staff is not uncommon in the region, but is thought to be deployed in Sierra Leone to a greater extent than elsewhere.^37^

Volunteer workers appear to be an important factor in the slow growth in the number of skilled health workers within Sierra Leone’s health workforce. The country has expanded its health sector payroll from approximately 10 000 to over 20 000^16 20^ between 2016–2022 and witnessed a significant increase in new health worker courses since 2019, which provides training for nursing and midwifery cadres that meet the WHO skilled-health worker definition. However, by creating an ever-expanding unsalaried health worker cohort, Sierra Leone’s authorities have raised expectations among these health workers of eventual payroll inclusion. It has put the awaiting unsalaried auxiliary staff in direct competition with new graduates who are higher skilled, to be hired when salaried posts come available. For MoH, every recruitment decision is a choice between improving the skilled-health worker to population ratio or honouring an unwritten contract with the volunteer health workforce which the health system continues to rely on. Between 2016 and 2019, a HRH inflection period in which policy on auxiliary cadres shifted, Sierra Leone’s MoH neglected to take the bold step to commit to upskilling all its pay-rolled health workers to the internationally defined minimum standard of ‘skilled’ health worker. This cemented the status of MCHAs and SECHNs as the preferred cadres for the majority of the country’s primary care provision, despite their limited training. It is a preference that continues until today, continuing to undermine Sierra Leone’s quality of care improvements efforts.

In the meantime, new graduates continue to take on volunteer roles. It is hard to know how many more volunteer health workers the public system can sustain, and how many new graduates will accept the prospect of years working as volunteers. To overcome its barriers to achieving UHC, Sierra Leone will need to embrace health workforce planning, create a multi-annual plan to gradually recruit both skilled and volunteer health workers and upskill all those on the payroll who do not meet ‘skilled’ health worker definition.

## Supplementary material

10.1136/bmjgh-2025-021043 online supplemental file 1

10.1136/bmjgh-2025-021043 online supplemental file 2

10.1136/bmjgh-2025-021043 online supplemental file 3

10.1136/bmjgh-2025-021043 online supplemental file 4

## Data Availability

Data are available in a public, open access repository.

## References

[1] United Nations (2015). The sustainable development goals. https://sdgs.un.org/goals.

[2] Hogan DR, Stevens GA, Hosseinpoor AR (2018). Monitoring universal health coverage within the Sustainable Development Goals: development and baseline data for an index of essential health services. Lancet Glob Health.

[3] World Health Organization (2023). WHO health workforce support and safeguards list 2023. https://www.who.int/publications-detail-redirect/9789240069787.

[4] Ng M, Fullman N, Dieleman JL (2014). Effective coverage: a metric for monitoring Universal Health Coverage. PLoS Med.

[5] Reid M, Gupta R, Roberts G (2020). Achieving Universal Health Coverage (UHC): Dominance analysis across 183 countries highlights importance of strengthening health workforce. PLoS ONE.

[6] Government of Sierra Leone, Ministry of Health and Sanitation (2021). National health and sanitation policy 2021.

[7] Government of Sierra Leone, Ministry of Health and Sanitation (2021). Universal health coverage roadmap for Sierra Leone 2021-2030.

[8] Rushton S (2005). Health and Peacebuilding: Resuscitating the Failed State in Sierra Leone. *International Relations*.

[9] Bertone MP, Witter S (2013). The development of HRH policy in Sierra Leone, 2002-2012-report on key informant interviews.

[10] Wurie H, Witter S, Raven J (2016). "Fighting a battle”: Ebola, health workers and the health system in Sierra Leone. www.rebuildconsortium.com.

[11] Dattani S, Spooner F, Ritchie H (2023). United nations inter-agency group for child mortality estimation. https://ourworldindata.org/grapher/child-mortality-by-income-level-of-country.

[12] Government of Sierra Leone, Ministry of Health and Sanitation (2017). Summary report of the 2017 SARA plus in Sierra Leone: service availability and readiness assessment (SARA), quality of care, and data quality review.

[13] P4H Network (2021). Meeting the African Union’s health spending standards to advance universal health coverage. https://p4h.world/en/countries/sierra-leone/.

[14] World Bank (2021). Sierra Leone public expenditure reviews 2021 - Improving quality of public expenditure in health. https://www.worldbank.org/.

[15] Government of Sierra Leone, Ministry of Finance (2024). A Citizen’s guide to the national budget 2024.

[16] Government of Sierra Leone, Ministry of Health and Sanitation (2021). National health sector strategic plan 2021-2025.

[17] Government of Sierra Leone, Ministry of Health and Sanitation (2017). National health sector strategic plan 2021-2025.

[18] Adegoke A, Utz B, Msuya SE (2012). Skilled Birth Attendants: who is who? A descriptive study of definitions and roles from nine Sub Saharan African countries. PLoS One.

[19] World Health Organisation (2018). Definition of skilled health personnel providing care during childbirth: the 2018 joint statement by WHO, UNFPA, UNICEF,ICM, ICN, FIGO and IPA. https://www.unfpa.org/sowmy.

[20] Government of Sierra Leone, Ministry of Health and Sanitation (2016). Directorate for human resources for health. Human resources for health country profile: Sierra Leone country profile.

[21] Government of Sierra Leone, Ministry of Health and Sanitation (2019). Republic of Sierra Leone health labour market analysis.

[22] Wurie HR, Samai M, Witter S (2016). Retention of health workers in rural Sierra Leone: findings from life histories. Hum Resour Health.

[23] Edoka I, Ensor T, McPake B (2016). Free health care for under-fives, expectant and recent mothers? Evaluating the impact of Sierra Leone’s free health care initiative. Health Econ Rev.

[24] Donnelly J (2011). How did Sierra Leone provide free health care?. Lancet.

[25] Jones SA (2020). Fragile states and the development of resilient health systems through the lens of human capital. Doctoral Thesis. http://eprints.hud.ac.uk/id/eprint/35405/.

[26] Braun V, Clarke V (2006). Using thematic analysis in psychology. Qual Res Psychol.

[27] Braun V, Clarke V, Weate P (2019). Reflecting on reflexive thematic analysis. Qual Res Sport Exerc Health.

[28] Sousa A, Scheffler RM, Nyoni J (2013). A comprehensive health labour market framework for universal health coverage. Bull World Health Organ.

[29] Statistics Sierra Leone (2022). 2021 mid-term population and housing census. http://www.statistics.sl/images/StatisticsSL/Documents/Census/MTPHC_Preliminary_Report/Final_Preliminary_Report_2021_MTPHC.pdf.

[30] Hudson B, Hunter D, Peckham S (2019). Policy failure and the policy-implementation gap: can policy support programs help?. Policy Design and Practice.

[31] Ahmat A, Okoroafor SC, Kazanga I (2022). The health workforce status in the WHO African Region: findings of a cross-sectional study. *BMJ Glob Health*.

[32] Tsawe M, Susuman AS (2022). Inequalities in maternal healthcare use in Sierra Leone: Evidence from the 2008-2019 Demographic and Health Surveys. PLoS ONE.

[33] Government of Sierra Leone, Ministry of Health and Sanitation (2017). Human resources for health policy 2017-2021.

[34] Goodman C, Witter S, Hellowell M (2024). Approaches, enablers and barriers to govern the private sector in health in low- and middle-income countries: a scoping review. *BMJ Glob Health*.

[35] Amri M, Sam O, Anye M (2025). The governance of private sector engagement in health in the African Region: a descriptive case study. Journal of Global Health Economics and Policy.

[36] Sumberg J, Fox L, Flynn J (2021). Africa’s “youth employment” crisis is actually a “missing jobs” crisis. Dev Policy Rev.

[37] Govindaraj R, Herbst CH, Clark JP (2018). Strenghening Post-EBOLA health systems. From response to resilience in Guinea, Liberia, and Sierra Leone.

