# Supplementary material for: Improving Sierra Leone’s skilled health-worker-to-population ratio: how unsalaried and auxiliary health workers are barriers in its path to universal health coverage
Source: BMJ Glob Health. 2025 Nov 17;10(11):e021043. doi: 10.1136/bmjgh-2025-021043 (PMC12636922; doi:10.1136/bmjgh-2025-021043)
Supplement: online supplemental file 1 [file bmjgh-10-11-s001.docx]

Supplementary file 1- Data collection sheet PHU staff

**Data collection sheet, salaried and unsalaried health workers at District level:**

District:________________________________________________________

Total # of PHUs:__________________________________________________

Total # CHCs_____________________________________________________

Total # CHPs_____________________________________________________

Total # MCHPs___________________________________________________

Total # hospitals (pls name, + indicate public, private, faith-based)___________________

________________________________________________________________

________________________________________________________________

Staffing table – PHUs, whole district:

|  | On payroll | Not on payroll* |
| --- | --- | --- |
| CHO |  |  |
| CHT |  |  |
| CHA |  |  |
| Midwives (incl. SECHN/ Midwives) |  |  |
| SRN |  |  |
| SECHN |  |  |
| MCH Aides |  |  |
| Nursing Aides |  |  |
| Lab Technicians |  |  |
| Auxiliary staff (cleaners, porters |  |  |
| Other |  |  |

*any staff on ‘post-basic’ unpaid work experience year can be counted as ‘not on payroll’.

Hospital staff (per hospital)___________________________________________________________

|  | On payroll | Not on payroll |
| --- | --- | --- |
| CHO |  |  |
| CHT |  |  |
| CHA |  |  |
| Midwives (incl. SECHN/ Midwives) |  |  |
| SRN |  |  |
| SECHN |  |  |
| MCH Aides |  |  |
| Nursing Aides |  |  |
| Lab Technicians |  |  |
| Auxiliary staff (cleaners, porters |  |  |
| Other |  |  |

Hospital staff (per hospital)___________________________________________________________

|  | On payroll | Not on payroll |
| --- | --- | --- |
| CHO |  |  |
| CHT |  |  |
| CHA |  |  |
| Midwives (incl. SECHN/ Midwives) |  |  |
| SRN |  |  |
| SECHN |  |  |
| MCH Aides |  |  |
| Nursing Aides |  |  |
| Lab Technicians |  |  |
| Auxiliary staff (cleaners, porters |  |  |
| Other |  |  |
