# Supplementary material for: Improving Sierra Leone’s skilled health-worker-to-population ratio: how unsalaried and auxiliary health workers are barriers in its path to universal health coverage
Source: BMJ Glob Health. 2025 Nov 17;10(11):e021043. doi: 10.1136/bmjgh-2025-021043 (PMC12636922; doi:10.1136/bmjgh-2025-021043)
Supplement: online supplemental file 2 [file bmjgh-10-11-s002.docx]

**Supplementary file 2-** **Data collection sheet,**

**health worker trainees per training institution**

Name of training institution:

Location:

Public/Private:

Number of trainees who started their training course, per year, per cadre:

|  | 2019 | 2020 | 2021 | 2022 | 2023 | 2024 | 2025 | 2026 |
| --- | --- | --- | --- | --- | --- | --- | --- | --- |
| Medical doctors |  |  |  |  |  |  |  |  |
| PA |  |  |  |  |  |  |  |  |
| CHO |  |  |  |  |  |  |  |  |
| CHT |  |  |  |  |  |  |  |  |
| CHA |  |  |  |  |  |  |  |  |
| Midwives |  |  |  |  |  |  |  |  |
| SECHN-Midwives |  |  |  |  |  |  |  |  |
| Public Health |  |  |  |  |  |  |  |  |
| BSc Nursing |  |  |  |  |  |  |  |  |
| SRN |  |  |  |  |  |  |  |  |
| SECHN |  |  |  |  |  |  |  |  |
| MCH Aides |  |  |  |  |  |  |  |  |
| Lab Technicians |  |  |  |  |  |  |  |  |
| Other: |  |  |  |  |  |  |  |  |
| Other: |  |  |  |  |  |  |  |  |
