# Supplementary material for: Improving Sierra Leone’s skilled health-worker-to-population ratio: how unsalaried and auxiliary health workers are barriers in its path to universal health coverage
Source: BMJ Glob Health. 2025 Nov 17;10(11):e021043. doi: 10.1136/bmjgh-2025-021043 (PMC12636922; doi:10.1136/bmjgh-2025-021043)
Supplement: online supplemental file 3 [file bmjgh-10-11-s003.docx]

Supplementary file 3: Good reporting of a mixed-methods study (GRAMMS) checklist

| Guidelines | Pages |
| --- | --- |
| 1. Describe the justification for using a mixed methods approach to the research question | 6-7 |
| 2. Describe the design in terms of the purpose, priority and sequence of methods | 6-7 |
| 3. Describe each method in terms of sampling, data collection and analysis | 6-7 |
| 4. Describe where integration has occurred, how it has occurred and who has participated in it | 6-7, 12 |
| 5. Describe any limitation of one method associated with the present of the other method | 14-15 |
| 6. Describe any insights gained from mixing or integrating methods | 15 |

Reference: O'Cathain A, Murphy E, Nicholl J. The quality of mixed methods studies in health services research. J Health Serv Res Policy. 2008;13: 92-98.
