# Supplementary material for: Improving Sierra Leone’s skilled health-worker-to-population ratio: how unsalaried and auxiliary health workers are barriers in its path to universal health coverage
Source: BMJ Glob Health. 2025 Nov 17;10(11):e021043. doi: 10.1136/bmjgh-2025-021043 (PMC12636922; doi:10.1136/bmjgh-2025-021043)
Supplement: online supplemental file 4 [file bmjgh-10-11-s004.docx]

Supplementary files 4**- Author Reflexivity Statement**

1. **How does this study address local research and policy priorities?**

The research focuses on the impact of unsalaried health workers who are clinically trained nurses, midwives or auxiliary nurses who have requested, upon graduation, and have been assigned a workplace by the local public health authorities or public hospital, without being added to the payroll. In 2016 an audit of human resources for health revealed that half of all public healthcare staff, approximately 36% of clinical staff, were deployed in this manner, without being on the payroll, without a salary, without insurance or any accountability. This research project examined the impact of the unsalaried health workers on healthcare delivery, by examining the coping strategies of unsalaried workers and by conducting research among the care-seeking population, to examine their views of their access to healthcare, in the context of Sierra Leone’s well-known fee-exemptions for primary healthcare for pregnant women and children under five. The researchers also examined the health worker training context and health workforce management policies and actions at MoH level. While health policy has stated since 2017 that it is the MoH’s objective to absorb all qualified health workers onto the payroll, our research demonstrated that the strategies contained within the national health strategic plan has not yet been implemented. As this affects all health worker trainees and all prospective patients, we believe that this is research addresses key local level concerns regarding healthcare access, and two key overarching policy priorities; improving health outcomes (which are affected by informal barriers to access to care) and achieving universal health coverage (which is affected by low health worker density).

1. **How were local researchers involved in study design?**

Despite having a small budget that covered only the cost of hiring the global north principal investigator, who was hired as a post-doc/ early career researcher and the PhD student (covering fees and a minimal stipend), this research team was able to make connections with a range of Sierra Leoneans who were involved in the research. Dr. DW, an independent researcher based in Freetown, was engaged as the sounding board for the research, she also managed the survey data collection. She commented on several drafts of research papers but does not want to be acknowledged as an author, due to her working relationship with MoH, who may interpret this study as negatively reflecting MoH’s work, and by association, may negatively affect her working relationship with MoH. In addition, the researchers engaged staff from the College of Medicine and Allied Health Sciences (COMAHS), who were interviewed as key informants and also invited to comment on the research findings. In addition, an Erasmus+ grant was obtained to facilitate a lecturing exchange for the PI to provide lectures at COMAHS and for one of the COMAHS teaching staff to travel to Ireland to lecture at the PI/PhD student’s university. We also reached out to researchers who work for a donor-funded private research institution and invited the researchers to several events, but despite showing an interest during electronic communication, the local researchers never made it to any of the Freetown-based in-person opportunities that we proposed to meet and discuss our study. Because of the lack of funding, there was no opportunity to recruit local researchers for the study. Instead, the small global north research team hired local translators in the three locations where the data collection took place. We have continued to engage with them after the initial data collection.

1. **How has funding been used to support the local research team?**

Because of the lack of funding, there was no opportunity to recruit local researchers for the study. Instead, the small global north research team hired local translators in the three locations where the data collection took place. We have continued to engage with them after the initial data collection by inviting them to dissemination events and by providing references and recommendations for subsequent employment. We were able to hire the two translators based in Freetown, who were in fact film makers, to create to short videos for the dissemination of the research findings of the study, thereby recognising their craft and providing a more rewarding project for them, after their short contribution to the research as translators.

1. **How are research staff who conducted data collection acknowledged?**

The research team has acknowledged the engagement of the translators and Dr DW in all of the publications. The researchers have conducted three findings sharing meetings, two in the capital Freetown and one in the District Head Quarters in Port Loko, to which all of the collaborating researchers and translators were invited, some of them attended, others were too busy with subsequent work. The Freetown-based translators were film makers who were engaged through a not-for-profit organisation that provides video and music production skills training for vulnerable youth. They have subsequently been hired as film makers to create two short videos that will be used to disseminate the research findings of the study. They are credited as the directors and film makers.

1. **Do all members of the research partnership have access to study data?**

Some, but not all, data is publicly available via an online data repository. Most interview date is sensitive and therefore not available to anyone other than the main researchers.

1. **How was data used to develop analytical skills within the partnership?**

As there were no early career researchers actively involved in working with the research team, this did not happen; both Dr DW and the researchers/lecturers at COMAHS were experienced academics, the translators had other day jobs as journalists and film makers.

1. **How have research partners collaborated in interpreting study data?**

Dr DW engaged in brainstorming and providing feedback on research articles, both in person and online. The COMAHS researchers collaborated as key informants. Local and international NGOs and aid donor agency staff were interviewed as key informants and most attended two-three findings validation/feedback sessions, which helped the authors to interpret the findings.

1. **How were research partners supported to develop writing skills?**

As there were no early career researchers actively involved in working with the research team, this did not happen; both Dr DW and the researchers, lecturers at COMAHS were experienced lecturers, the translators were not academically engaged.

1. **How will research products be shared to address local needs?**

The research findings have been shared several times with a range of local stakeholders, including aid donors and NGOs who are probably best placed to make use of the data. So far, one NGO has used our findings for an advocacy report, while another is using findings for a campaign to create more access to maternal health. The PhD student created a short audio file with snippets of interview and ambient sound recordings (with consent) that raised awareness of the issues that were uncovered during the study, which was aired on local radio. In addition, the videos that were being produced will also be circulated at local level (a one-minute version is being developed for local social media), all containing key advocacy messages that contain a call for MoH to implement the human resources for health policies it has formulated and published, as this would serve local needs most.

**10. How is the leadership, contribution and ownership of this work by LMIC researchers recognised**

**within the authorship?**

This has only been partly achieved, due to the fact that the Sierra Leonean academic who contributed most to this study, prefers to remain anonymous, as this is better for her continued job prospects.

**11. How have early career researchers across the partnership been included within the authorship**

**team?**

The only early career researcher who has been engaged in this study is the PhD student, who has been listed as the second author on all published papers and who has presented his findings alongside the PI on many occasions. The PI has deliberately not foregrounded the findings of the PhD student in any of the publications, as the PhD student aims to publish his findings in a publication where he will be the first author, as soon as he has completed writing his thesis.

**12. How has gender balance been addressed within the authorship?**

The PI is female, while the PhD student is male. Our most important research collaborator, Dr DW is female, while the main researchers and academics at COMAHS (including the academic who took part in the Erasmus exchange) were male, reflecting the male dominance of the senior academic staff at COMAHS. Five out of the six translators were female, we deliberately sought to recruit females as translators, as the main focus of the research were also female, both health workers and prospective patients who were entitled to fee exemptions for maternal and child health.

**13. How has the project contributed to training of LMIC researchers?**

Unfortunately, there was no funding for the hiring and training of LMIC researchers. The research funding covered only the cost of the PI, who was not in tenured employment, and the PhD student, based on EU fees and stipend. The only training of LMIC researchers that happened during this research project was the lecturing that the PI did during her week providing lectures at COMAHS to nursing undergraduate and post-graduate students.

**14. How has the project contributed to improvements in local infrastructure?**

The research has not contributed to improvements of local infrastructure, but it has updated the local knowledge regarding the total percentage of informally deployment of unsalaried clinical health workers in public health facilities (which was last counted in 2016 and has now been updated – primary care level only), a practice that results in labour exploitation and barriers to access to health.

**15. What safeguarding procedures were used to protect local study participants and researchers?**

All research participants were given a verbal explanation about the research in their own language, and a written copy of the same information in English, with contact details of the researchers and the local research ethics authorities. All participants were given the option to participate in the research and to sign a consent form. All participants were told that they could opt out of participating at any time. All participants were interviewed anonymously, no names were recorded from any of the health worker and community interviewees. Key informant interviewees were acknowledged by their job description. All audio recordings of the interviews were uploaded on a secure server and they will be kept for the least possible amount of time, before being destroyed. We only had one local researcher who supported the research, but was not involved in face-to-face data collection. The only action we took to safeguard her, was to not name her as an author in any of our research publications.
